# Case report: Anesthesia management for surgical treatment of glucagonoma with symptom of characterized necrolytic migratory erythema

**DOI:** 10.3389/fonc.2024.1408506

**Published:** 2024-12-19

**Authors:** Di Xia, Le Shen

**Affiliations:** Department of Anesthesiology, Peking Union Medical College Hospital, Beijing, China

**Keywords:** glucagonoma, anesthesia management, necrolytic migratory erythema, fiberoptic intubation, difficult airway

## Abstract

**Background:**

The anesthetic management of patients with glucagonoma is complicated by a number of factors including glucose fluctuation, characterized necrolytic migratory erythema in oral and pharyngeal, which may lead to an unexpected difficult airway.

**Case presentation:**

Herein we describe the anesthetic considerations and management of a 47-year-old adult with glucagonoma, who presented for a laparoscopic splenectomy and distal pancreatectomy procedure.

**Conclusion:**

This report details fiberoptic intubation in an adult with glucagonoma and necrolytic migratory erythema. We recommend that this approach be considered in patients with glucagonoma and severe necrolytic migratory erythema undergoing general anesthesia.

## Background

Glucagonoma is a rare pancreatic islet cell tumor in clinical practice, characterized by symptoms such as necrolytic migratory erythema, diabetes, and hypoglycemia (1). Necrolytic migratory erythema is the primary onset characteristic in 70–80 percent of patients (2). Within 1-2 weeks, this erythema typically spontaneously gets worse (3). Clinical signs of airway ulcers in certain people include glossitis or stomatitis. The best treatment for this illness right now is surgical excision of the tumor (4). The management of blood glucose changes during surgery and the protection of the face and unexpected difficult airway during induction and intubation are significant obstacles to anesthetic management.

## Case

A 47-year-old man, complaining of wandering erythema all over his body for more than two years, came to the hospital for treatment. The patient experienced symmetrical red papules on the front chest and back two years ago, but there was no apparent explanation and they were accompanied by itching. The patient experienced symmetrical red papules on the front chest and back two years ago, but there was no apparent explanation and they were accompanied by itching. The patient ignored them, though. One month later, the same type of rash reappeared on both legs, the waist, and the buttocks. The local hospital diagnosed it as “eczema” and prescribed anti-inflammatory medications, Chinese patent medicines, topical antifungal medications, etc., but the results were poor and the symptoms gradually grew wider. The patient’s vulva, hands, feet, and face all eventually developed edematous erythematous and blisters that eventually burst and inflicted excruciating pain. Methylprednisolone was once prescribed at the community hospital, but the results were not excellent. Since beginning, the patient’s has shed 8 kg of weight. During the disease, there were intermittent dry mouth, dry tongue, and oral ulcers, but no fever, abdominal pain, diarrhea, nausea, or vomiting. The patient denied other clinical symptoms, was usually healthy, and denied other comorbidities and complications.

**Figure 1:**
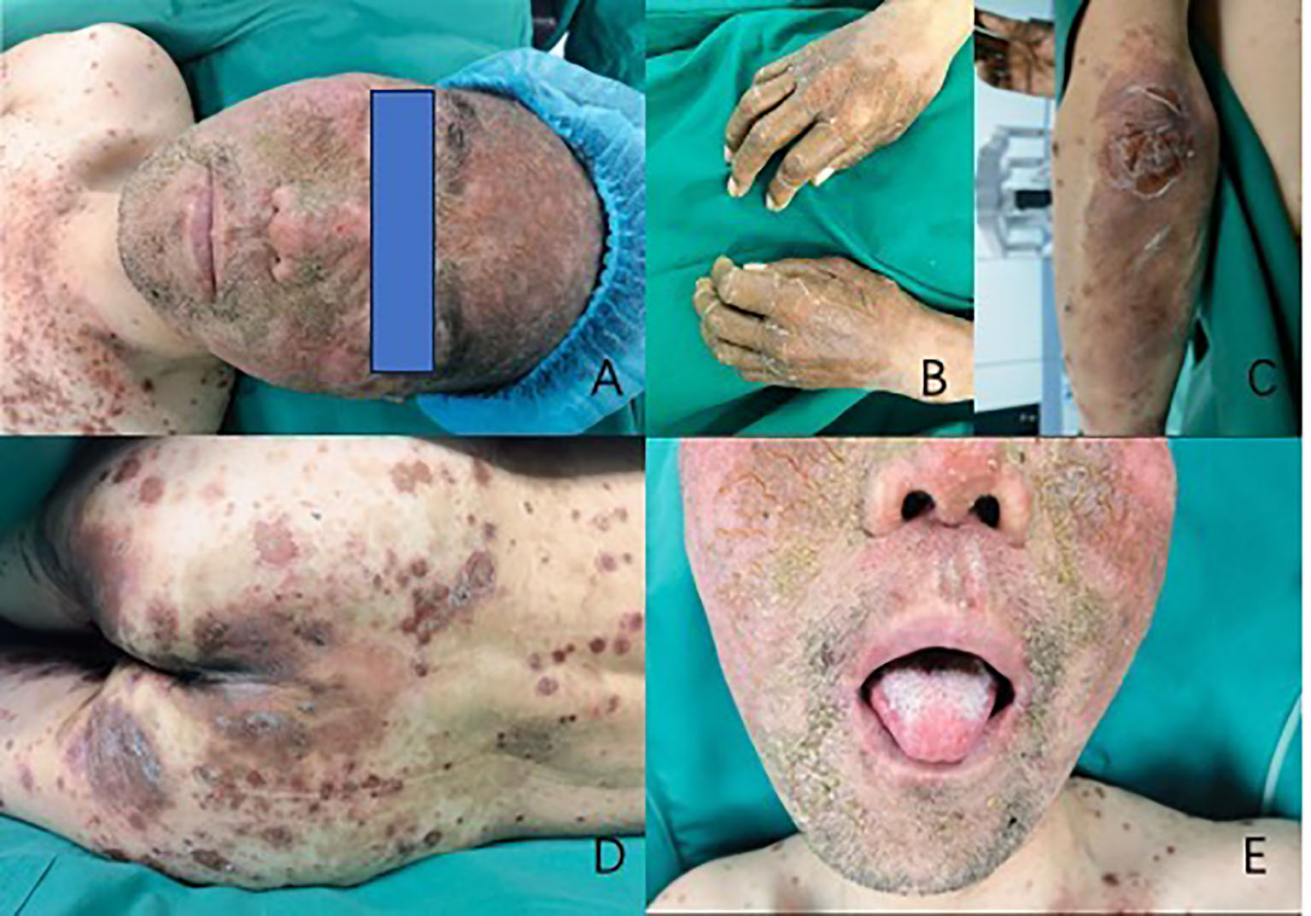
Cutaneous manifestations of necrolytic migratory erythema. Necrolytic migratory erythema distributed on the patient’s face **(A)**, hands **(B)**, elbows **(C)**, and sacrum **(D)**. Figure **E** shows glossitis, stomatitis, or cheilitis.

Physical examination reveals that the patient has facial erythema, maculopapular lesions on the trunk, vulva, and dorsum of the hands and feet, some of which are breaking down and crusting, dense red papules, scattered edematous lesions, and erythema. The tongue is red and exhibits inflammation-related symptoms (**Figure 1**).

**Figure 2:**
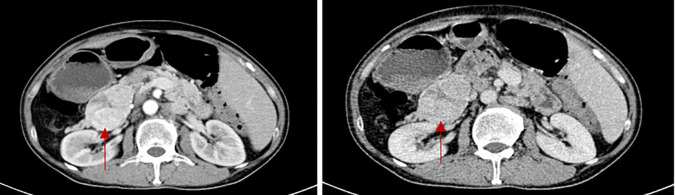
The CT scan of glucagonoma (The red arrow shows the location of the tumor. The two images show the arterial phase of the enhanced CT scan and the portal phase of the enhanced CT scan.)

Laboratory testing revealed his glucagon was 536pg/ml (normal: 0-200). Patients were also measured for insulin levels(insulin 18.9μIU/mL), ACTH level(ACTH 74.6pg/ml), and total blood cortisol levels(F 29.3μg/dl), all above tests help to exclude the possibility of combining other endocrine tumors. The abdominal pelvic enhanced CT revealed a mixed density mass in the body and tail of the pancreas measuring roughly 6.2 cm 4.3 cm with punctate calcification at the edge (**Figure 2**). The arterial phase showed obvious uneven enhancement and the portal vein phase was still slightly higher than normal parenchyma. The octreotide and SPECT/CT fusion imaging examination suggested a soft tissue density mass in the tail of the pancreas with high expression of somatostatin, suggesting a possible neuroendocrine tumor. Complete histological analysis: apoptosis of spinous cells above the spinous layer, considering necrotic migratory erythema. It is considered that the diagnosis of pancreatic neuroendocrine tumor in the patient is clear and there is a high possibility of glucagonoma.

**Figure 3:**
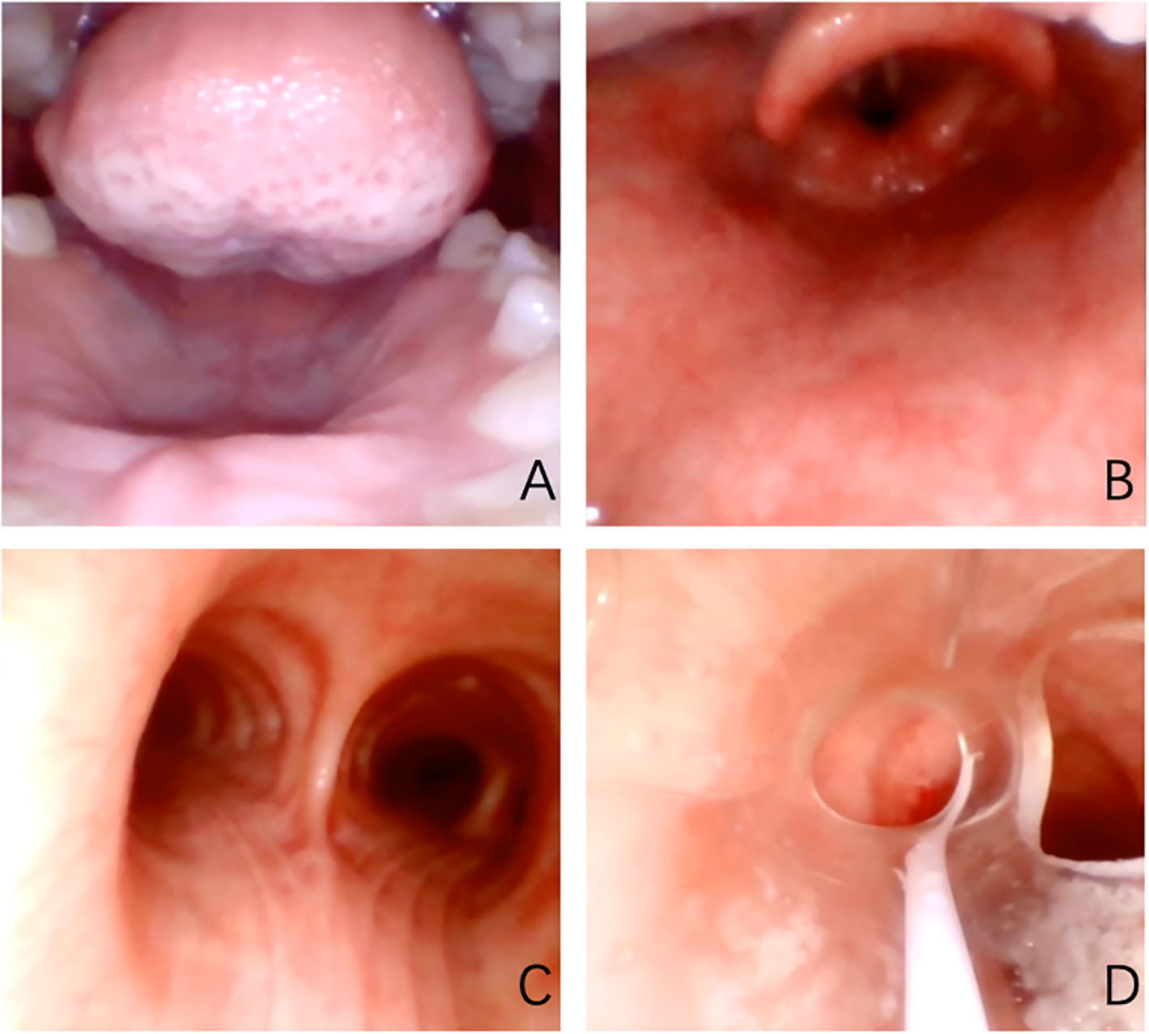
Oropharyngeal cavity under fiberscope. **(A)** shows the narrow pharynx; **(B)** shows glottis under fiberoptic bronchoscope; **(C)** shows the carina; **(D)** shows a tracheal catheter placed in the appropriate position within the airway.

**Figure 4:**
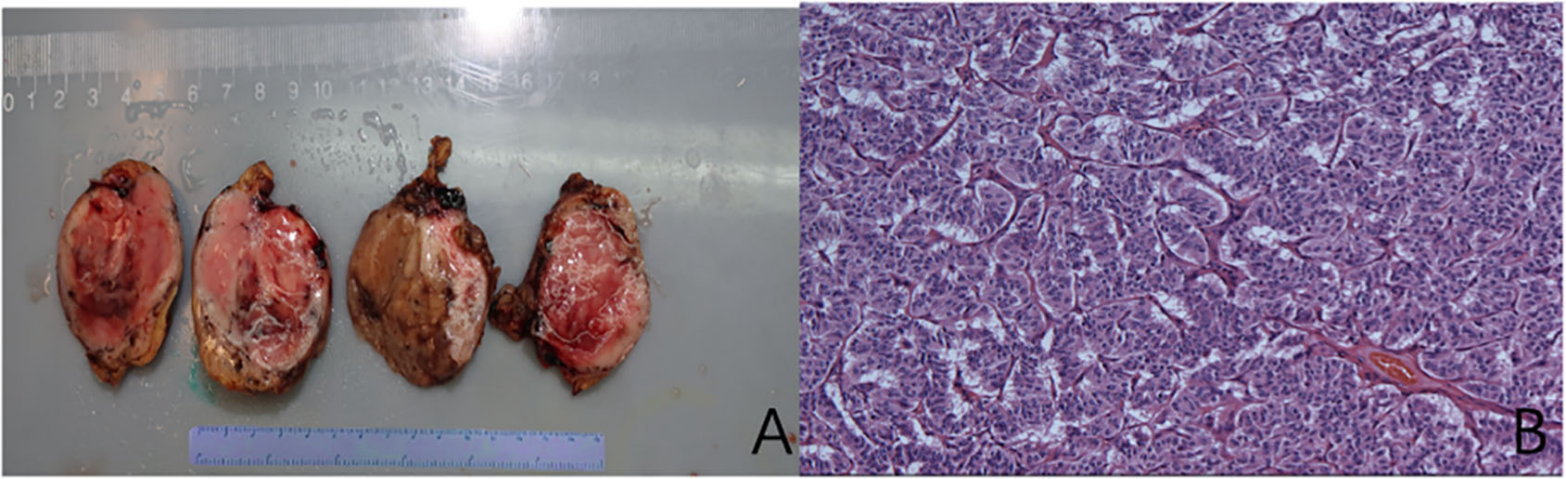
Pancreatic glucagonoma tumor body **(A)**; Histological examination of the mass showing an **(B)** (Hematoxylin and Eosin (H&E) staining of tissue section, showing well-defined alpha-cell pancreatic tumor cellular architecture).

The patient was hospitalized to the general surgery unit and scheduled for a laparoscopic splenectomy and distal pancreatectomy. The patient’s airway condition was reevaluated after he was brought into the operating room. His mouth could only open up to less than three transverse fingers because of the erythema, dermatitis, and scabs on his face and mouth. To avoid injury and bleeding to the patient’s face, the plan is to use general anesthesia, with rapid sequence induction and fiberoptic-guided endotracheal intubation. Anesthesia was induced with propofol 100mg and sufentanil 10ug, and rocuronium was given to facilitate endotracheal intubation and intraoperative muscle relaxation. After the patient fell asleep, the fiberoptic bronchoscope was inserted into the patient’s mouth and it was found that the mucous membrane inside the patient’s mouth was swollen and congested, with surrounding tissue proliferation and a constricted narrow pharynx. (**Figure 3A**) The fiberoptic bronchoscope was gently inserted along the patient’s tongue root, entered the laryngopharynx, and saw the glottis. (**Figure 3B**) It was gently passed through the glottis and continued to be inserted downward to see the carina. (**Figure 3C**) The fiberoptic bronchoscope was fixed and guided the smooth insertion of the tracheal catheter and then withdrew the fiberoptic bronchoscope. (**Figure 3D**) Anesthesia was maintained with isoflurane 2% and was maintained in mac value 0.8-1, supplemented with a total of 40ug sufentanil. His blood sugar level was 5.6mmol/l before surgery and was monitored during surgery (**Table 1**). The surgeon was successful in completely removing the body and tail of the pancreas and the spleen, and cleaning the lymph nodes around the pancreas and beside the superior mesenteric artery (**Figure 4A**). Neuromuscular blockade was reversed with neostigmine and atropine. The patient emerged from general anesthesia and had the tracheal tube removed uneventfully. The postoperative retest of glucagon dropped to 70pg/mL, and the patient was discharged on the eighth day after surgery. The removed pancreatic tumor and lymph nodes were sent to the pathology department for examination. Postoperative pathological examination confirmed the diagnosis of glucagonoma (**Figure 4B**), and immunohistochemical staining revealed positive staining for chromogranin A(CgA), synaptophysin (Syn), and Ki-67(index 8%) (**Figure 4B**), with no lymph node metastasis.

**Table 1:** Alterations in the patient's blood glucose.

Time	Glu (mmol/l)
Before surgery	5.4
Beginning of surgery	4.1
After tumor removal	6.4
Ending of surgery	5.7
30min after surgery	3.5
1 day after surgery	12.1
3 day after surgery	5.6

## Discussion

Glucagonoma is a rare pancreatic endocrine tumor with an annual incidence of 0.01/1 million to 0.1/1 million. According to reports, the median age of patients diagnosed with insulinoma syndrome is 53.3 years old, and the average time from the onset of symptoms to a definitive diagnosis is 39 months (5). Due to the non-specific clinical features of glucagonoma, many patients are diagnosed only after the tumor has metastasized (3). The main clinical manifestations of glucagonoma include impaired glucose tolerance or diabetes, necrolytic migratory erythema (NME), glossitis, stomatitis, cheilitis, chronic diarrhea, venous thrombosis, dilated cardiomyopathy, and mental symptoms (6). 70-80% of patients have NME as the characteristic feature of the disease (3). NME probably results from hyponutrition and amino acid deficiency (7). Initially, it is erythematous vesicles and bullae. After rupture, it spreads outward with crusting and pigmentation. The rash is sometimes the only symptom and most commonly occurs on the face, perineum, and limbs. Within 7-14 days, the skin lesions enlarge and merge, often accompanied by itching and pain (8). 41% of patients report glossitis, stomatitis, or cheilitis, and the mucosal lesion process is similar to that of the skin (3). The diagnosis of NME is mainly based on skin biopsy. Pathology shows epidermal laminar necrosis and separation of the outer layer of the epidermis with perivascular lymphocyte and histiocyte infiltration. The skin biopsy of this patient supports the pathological diagnosis of NME. If the patient has typical NME, weight loss, elevated fasting blood sugar, chronic diarrhea, deep vein thrombosis, etc., a pancreatic glucagonoma should be highly suspected. The basis for a clear diagnosis is an increase in the patient’s fasting plasma glucagon level. Currently, the diagnostic criterion is mostly a fasting plasma glucagon level exceeding 500ng/L (9). Surgery, chemotherapy and hormone therapy such as somatostatin are the main treatments for this disease, among which surgical treatment is the most thorough means (9).

For patients with glucagonoma undergoing surgical anesthesia management, the changes in blood glucose should be monitored at all times during the operation. When the tumor is stripped, especially before vascular ligation, there may be a high peak of glucagonoma secretion in the blood, which may have adverse effects on blood glucose homeostasis and the internal environment. The duration of the operation was 245 minutes. The highest peak of blood glucose during the operation was 120 minutes after the start of the operation (i.e. when the tumor was removed), with a peak value of 6.4mmol/l. The main reason for its increase may be due to the squeezing of the tumor during the operation, or it may be surgical stress. The patient’s blood sugar dropped to 3.5mmol/l after returning to the ward for 2 hours, and glucose was supplemented in the ward. At this time, it was about 2.5 hours after the tumor was removed, indicating that the most obvious period of blood sugar decline in such patients may occur 1-3 hours after tumor removal, and multiple case reports have also reached similar conclusions (10). At this time, attention should be paid to changes in blood sugar, and glucose should be actively infused if necessary to prevent hypoglycemic coma. On the first day after the operation, the re-examination of glucagon also dropped from 536pg/ml before the operation to the normal value of 184 pg/ml. This also confirmed that the operation successfully removed the glucagonoma.

Clinically, pharmaceutical amounts of glucagon can not only elevate blood glucose but also increase cardiac contraction by increasing the amount of cyclic adenosine monophosphate present in myocardial cells. However, in this case, there was no obvious change in the patient’s cardiovascular system. The explanation might be that the clinical application dose of glucagon in acute heart failure is 50-70 μg/kg, and the average adult loading dose is 3.5 mg, while the increase in glucagon during the operation did not reach this pharmacological activity level. In order to reduce the patient’s surgical stress and safeguard normal glucose metabolism in order to maintain endocrine stability, we also employed sevoflurane inhalation anesthetic in addition to sufentanil combined with remifentanil intravenously for analgesia throughout the procedure. To a certain extent, it can alleviate intraoperative blood glucose elevation and violent fluctuations in circulation. Additionally, sufentanil and remifentanil have also been proven to have good myocardial protective effects (11, 12). Some case reports recommend general anesthesia combined with epidural anesthesia to stabilize the circulation in patients with glucagon tumors. However, the inhalation anesthetic utilized in this case report was coupled with intravenous remifentanil and sufentanil to guarantee hemodynamic stability. Additionally, epidural puncture insertion is likely to result in additional damage and an increased risk of infection in individuals with necrolytic loosening wandering erythema. In this case, the patient’s use of intermittent single administration of sufentanil combined with continuous infusion of remifentanil also played a positive role in cardiac events.

In addition, it has been reported that 41% of patients develop glossitis, stomatitis, or cheilitis, with a higher incidence in women, with an incidence of 42.4% and a female incidence of 67.3% (3). Patients with glossitis and stomatitis mostly have congestion mild edema in the mouth and pharynx, and even rash and ulceration. Therefore, protection of the face and upper airway during anesthesia is also crucial. Previous case reports did not provide detailed information on how to protect the facial and intraoral structures during anesthesia (13). In this case, the patient’s intubation method used bronchoscope-guided endotracheal intubation to avoid damage to the oropharynx caused by laryngoscope insertion, and at the same time can observe and monitor the patient’s upper airway lesions in real-time. This patient had stomatitis with congestion and edema, but bronchoscopy showed that the tissue structure of the airway below the glottis was normal and no obvious lesions were found. In the end, the patient successfully underwent endotracheal intubation without aggravating injuries to the face and oral mucosa.

Glucagonoma may be part of multiple endocrine neoplasia syndrome (14, 15). Before the operation, the levels of serum calcium, parathyroid hormone, growth hormone, and other hormones should be actively measured to screen for the presence of pituitary tumors and parathyroid tumors to exclude the possibility of multiple endocrine neoplasia.

Glucagonoma is a rare special endocrine tumor with typical manifestations of NME, abnormal glucose tolerance, stomatitis, and glossitis. Its anesthesia management requires close monitoring of perioperative blood glucose and circulatory changes and attaches great importance to blood glucose and circulatory fluctuations caused by glucagon release. For cardiovascular events, sufficient sedation and analgesia should be used to inhibit stress response and reduce the pharmacological effects of glucagon on the myocardium. For patients with stomatitis and glossitis, airway lesions and their protection should be fully considered to avoid secondary damage and improve the prognosis and quality of life of patients.

## Data Availability

The original contributions presented in the study are included in the article/supplementary material. Further inquiries can be directed to the corresponding author.

## References

[1] Batcher E Madaj P Gianoukakis AG . Pancreatic neuroendocrine tumors. Endocr Res. (2011) 36:35–43. doi: 10.3109/07435800.2010.525085 21226566

[2] Li W Yang X Deng Y Jiang Y Xu G Li E . Dong S et al: Necrolytic migratory erythema is an important visual cutaneous clue of glucagonoma. Sci Rep. (2022) 12:9053. doi: 10.1038/s41598-022-12882-2 35641533 PMC9156669

[3] Song X Zheng S Yang G Xiong G Cao Z Feng M . Glucagonoma and the glucagonoma syndrome. Oncol Lett. (2018) 15:2749–55. doi: 10.3892/ol.2017.7703 PMC5778850 29435000

[4] Phan GQ Yeo CJ Hruban RH Lillemoe KD Pitt HA Cameron JL . Surgical experience with pancreatic and peripancreatic neuroendocrine tumors: review of 125 patients. J Gastrointest Surg. (1998) 2:472–82. doi: 10.1016/S1091-255X(98)80039-5 9843608

[5] Makis W McCann K Riauka TA McEwan AJ . Glucagonoma pancreatic neuroendocrine tumor treated with 177Lu DOTATATE induction and maintenance peptide receptor radionuclide therapy. Clin Nucl Med. (2015) 40:877–9. doi: 10.1097/RLU.0000000000000891 26204206

[6] Doherty GM . Rare endocrine tumours of the GI tract. Best Pract Res Clin Gastroenterol. (2005) 19:807–17. doi: 10.1016/j.bpg.2005.05.004 16253902

[7] Norton JA Kahn CR Schiebinger R Gorschboth C Brennan MF . Amino acid deficiency and the skin rash associated with glucagonoma. Ann Intern Med. (1979) 91:213–5. doi: 10.7326/0003-4819-91-2-213 111595

[8] Wilkinson DS . Necrolytic migratory erythema with carcinoma of the pancreas. Trans St Johns Hosp Dermatol Soc. (1973) 59:244–50. 4793623

[9] Kanakis G Kaltsas G . Biochemical markers for gastroenteropancreatic neuroendocrine tumours (GEP-NETs). Best Pract Res Clin Gastroenterol. (2012) 26:791–802. doi: 10.1016/j.bpg.2012.12.006 23582919

[10] Cao X Wang X Lu Y Zhao B Shi J Guan Q . Spleen-preserving distal pancreatectomy and lymphadenectomy for glucagonoma syndrome: A case report. Med (Baltimore). (2019) 98:e17037. doi: 10.1097/MD.0000000000017037 PMC6756711 31567941

[11] Zang H Shao G Lou Y . Sufentanil alleviates sepsis-induced myocardial injury and stress response in rats through the ERK/GSK-3beta signaling axis. Evid Based Complement Alternat Med. (2022) 2022:9630716. doi: 10.1155/2022/9630716 35774755 PMC9239792

[12] Kato R Foex P . Myocardial protection by anesthetic agents against ischemia-reperfusion injury: an update for anesthesiologists. Can J Anaesth. (2002) 49:777–91. doi: 10.1007/BF03017409 12374705

[13] Gin VC Zacharias M . Glucagonoma: anaesthetic management. Anaesth Intensive Care. (2009) 37:329–30. 19400510

[14] Brandi ML Agarwal SK Perrier ND Lines KE Valk GD Thakker RV . Multiple endocrine neoplasia type 1: latest insights. Endocr Rev. (2021) 42:133–70. doi: 10.1210/endrev/bnaa031 PMC7958143 33249439

[15] Niederle B Selberherr A Bartsch DK Brandi ML Doherty GM Falconi M . Multiple Endocrine Neoplasia Type 1 and the Pancreas: Diagnosis and Treatment of Functioning and Non-Functioning Pancreatic and Duodenal Neuroendocrine Neoplasia within the MEN1 Syndrome - An International Consensus Statement. Neuroendocrinology. (2021) 111:609–30. doi: 10.1159/000511791 32971521

